# Hyperspectral Estimation Models of Winter Wheat Chlorophyll Content Under Elevated CO_2_

**DOI:** 10.3389/fpls.2021.642917

**Published:** 2021-03-25

**Authors:** Yao Cai, Yuxuan Miao, Hao Wu, Dan Wang

**Affiliations:** Department of Ecology, College of Applied Meteorology, Nanjing University of Information Science and Technology, Nanjing, China

**Keywords:** elevated CO_2_, hyperspectral estimation model, chlorophyll content, red edge position, sensitive band, spectral index, winter wheat

## Abstract

Chlorophyll content is an important indicator of winter wheat health status. It is valuable to investigate whether the relationship between spectral reflectance and the chlorophyll content differs under elevated CO_2_ condition. In this open-top chamber experiment, the CO_2_ treatments were categorized into ambient (aCO_2_; about 400 μmol⋅mol^–1^) or elevated (eCO_2_; ambient + 200 μmol⋅mol^–1^) levels. The correlation between the spectral reflectance and the chlorophyll content of the winter wheat were analyzed by constructing the estimation model based on red edge position, sensitive band and spectral index methods, respectively. The results showed that there was a close relationship between chlorophyll content and the canopy spectral curve characteristics of winter wheat. Chlorophyll content was better estimated based on sensitive spectral bands and difference vegetation index (DVI) under both aCO_2_ and eCO_2_ conditions, though the accuracy of the models varied under different CO_2_ conditions. The results suggested that the hyperspectral measurement can be effectively used to estimate the chlorophyll content under both aCO_2_ and eCO_2_ conditionsand could provide a useful tool for monitoring plants physiology and growth.

## Introduction

It is expected that the atmospheric CO_2_ concentration will rise to 550 μmol⋅mol^–1^ in 2050 and reach or exceed 700 μmol⋅mol^–1^ at the end of the 21st century due to the increase of human population, energy production and utilization, deforestation and other intensive human activities (IPCC, 2013). Wheat is one of the world’s most productive and important crops in the 21st century, and also the main source of food for human (Curtis and Halford, 2014). Under elevated CO_2_, the physiology, growth and yield of wheat and other species are affected (Long et al., 2006; Wang et al., 2012).

Chlorophyll content was closely related to crop health, photosynthetic capacity and crop yield (Lukas et al., 2014). C_3_ plants are more sensitive to elevated CO_2_ than C_4_ plants (Leakey et al., 2009). The chlorophyll content and photosynthetic rate of varieties of C_3_ species, including crops and trees, was increased by elevated CO_2_ (Zhang et al., 2013; Madhana et al., 2014; Fathurrahman et al., 2016; Choi et al., 2017). For wheat, previous studies had shown a positive (Dubey et al., 2015) or negative (Wang et al., 2013) CO_2_ effects on the chlorophyll content and the difference might be resulted from the different experimental settings or CO_2_ increasing levels used in different studies. Given that systematic measurement of chlorophyll contents in elevated CO_2_ condition is scarce, a detailed measurement of chlorophyll content of winter wheat throughout the growing season will be useful to understand the effect of elevated CO_2_ on the physiology and growth of winter wheat.

Remote sensing methods could be used to accurately and rapidly relate variations in leaf optical properties with important plant characteristics, such as chlorophyll content and photosynthetic properties at the leaf and canopy scales (Ainsworth et al., 2014). Inversion of chlorophyll content by hyperspectral remote sensing was of great significance for crop growth status monitoring, yield estimation and agricultural planning (Liang et al., 2012; Flores-De-Santiago et al., 2013). Hyperspectral remote sensing had been used to monitor winter wheat chlorophyll content (He et al., 2018; Kasim et al., 2018). However, the application had been limited to specific test conditions (Serbin et al., 2012; Zhou et al., 2016) and there were few studies investigating hyperspectral remote sensing applications on winter wheat under elevated CO_2_ conditions.

The hyperspectral estimation models could be determined through different techniques to extract hyperspectral characteristics, including reflectance spectrum and first derivative spectrum, absorption and reflection location and vegetation index (Li et al., 2014). Previous research studied the relationship between visible and near-infrared spectra and leaf chemical components and found out that the original spectral reflectance and the first and second derivatives of the spectra could be used to estimate crop agronomic parameters (Card et al., 1988). Red edge and sensitive bands based spectral models had been used to simulate chlorophyll and nitrogen content of many species (Hansen and Schjoerring, 2003; Chen et al., 2013; Clevers and Gitelson, 2013; Stratoulias et al., 2015). Identifying optimal hyperspectral estimation models of winter wheat under different CO_2_ conditions is critical in crop growth monitoring and forecasting and requires further investigation.

In order to find an optimal estimation model for chlorophyll content and promote spectral analysis in the application of agriculture management under future global change conditions, an open top chamber (OTC) based CO_2_ manipulation experiment was conducted for 2 years in this study. The objectives of this study were: (1) to establish statistical models to study the relationship between hyperspectral characteristics and chlorophyll content of winter wheat throughout the growing stages; (2) to investigate whether the relationship between hyperspectral characteristics and chlorophyll content varies under elevated CO_2_ conditions.

## Materials and Methods

### Experimental Site

The study site was located in the agrometeorological experimental station of Nanjing University of Information Science and Technology, in Nanjing city, Jiangsu province of China (32°16’N, 118°86’E). The climate in this region characterizes subtropical monsoon season, with annual average precipitation of 1,100 mm, the average temperature in recent years of 15.6°C and the average annual frost-free period of 237 days. The soil texture in the tillage layer of winter wheat was loamy clay, and the clayey content was 26.1%. The bulk density of 0–20 cm soil was 1.57 g⋅cm^–3^, the pH (H_2_O) value was 6.3, and the organic carbon and total nitrogen content were 11.95 and 1.19 g⋅kg^–1^, respectively.

### Experimental Design

Open top chambers (OTC) were used in the experiment to manipulate CO_2_ concentration. There were eight OTC chambers, all of which were octagonal prisms (opposite side diameter 3.75 m, height 3 m, bottom area 10 m^2^) and equipped with aluminum alloy frames and toughened glass with high transmittance. There were two CO_2_ treatments, ambient CO_2_ (aCO_2_) and elevated CO_2_ (eCO_2_, aCO_2_ + 200 μmol⋅mol^–1^), each with four replicates. The treatment of elevated CO_2_ started from regreening stage and lasted to the end of growing stage.

In order to avoid the rapid loss of CO_2_ gas and reduce the experiment cost, the top opening of OTC was designed to tilt inward for 45°. The CO_2_ concentration in the chambers was controlled with an automatic control platform, composed of CO_2_ sensors, gas-supplying devices and automatic control system. Three wind-blowing fans were placed in each chamber to make the CO_2_ gas in the chamber evenly distributed. The CO_2_ sensor feeds back the CO_2_ concentration information in the chamber to the automatic control system every two seconds. The CO_2_ concentration averaged was 650 ± 58 μmol⋅mol^–1^ in elevated CO_2_ chambers and 455 ± 42 μmol⋅mol^–1^ in ambient chambers across two growing seasons.

The local winter wheat variety of Ningmai 13 was selected in the study. The field measurement of spectrum and chlorophyll was conducted in 2018–2019 and 2019–2020 growing seasons. During the whole growing stages, fertilizer and water management were carried out in the local conventional way.

### Spectrum Measurement

The spectral reflectance of winter wheat was measured by Field Spec4 of American analytical spectral device (ASD). The wavelength range was set at 350–2,500 nm. The sampling interval and resolution was set at 1.4 and 3 nm in the range of 350–1,000 nm; and 2 and 10 nm in 1,001–2,500 nm, respectively. The reflectance of winter wheat at five growth stages (jointing, booting, heading, filling and maturity stage) was measured on sunny days at 10:00 a.m.–2:00 p.m. Field Spec4 needed to be preheated 30 min before measurement. During the measurement, the sensor probe was placed vertically downward, the field of view angle was 10° and the probe was about 20 cm away from the top of the canopy. The measurement was carried out 10 times in different areas of an OTC. The reference white board was corrected immediately before and after the measurements in each chamber.

### Measurement of Chlorophyll Content

At the same time as the spectral measurement, the chlorophyll content was measured by the portable chlorophyll meter SPAD-502. Relevant studies have shown that soil and plant analyzer developrnent (SPAD) value was positively correlated with the total chlorophyll content, with the correlation coefficient up to 0.99, and the SPAD value could be used to represent the chlorophyll content of plants (Costa et al., 2001; Uddling et al., 2007). When measuring the chlorophyll content, five wheat plants were selected at the corresponding position of canopy spectrum measurement, then SPAD values were measured for five times uniformly on the upper, middle and lower leaves of each plant, and the average value was taken as the chlorophyll content of this sample point. A total of 200 chlorophyll samples were measured in 2018–2019 and 120 samples in 2019–2020. Two years of data were combined together, among which 240 samples were selected to establish the models, and the remaining 80 samples were used to verify the models.

### Statistical Analyses

View Specpro_6.0, Matlab_2017 and Origin_2018 were used to process and analyze the data. The spectral band range was set at 350–1,350 nm, and the wavelength corresponding to the largest first-order differential value in the red edge range (680–760 nm) was selected as the red edge position λr. The correlation analysis between canopy spectral reflectance and SPAD values of winter wheat was conducted, and the correlation coefficient was calculated to find out the sensitive bands. According to the original reflectance of winter wheat canopy, five common vegetation indexes were calculated. Each vegetation index had different characteristics. The normalized difference vegetation index (NDVI) was a common vegetation index and very sensitive to green vegetation. Ratio vegetation index (RVI) was sensitive to vegetation with high coverage. Difference vegetation index (DVI) and perpendicular vegetation index (PVI) were sensitive to the change of soil background. Optimizing soil and adjusting vegeta${\hat{y}}_{\text{i}}$tion index (OSAVI) explained the changes in the optical characteristics of the background and corrected the sensitivity of NDVI to the soil background (Bannari et al., 2007; Yan et al., 2013). The calculation of each vegetation index was listed in Table 1.

**TABLE 1:** The calculation of the vegetation indexes.

Spectral index	Formulation	Authors
NDVI	*NDVI* = (*R*_*NIR*_−*R*_*RED*_)/(*R*_*NIR*_ + *R*_*RED*_)	Rouse et al., 1973
RVI	*RVI* = *R*_*NIR*_/*R*_*RED*_	Jordan, 1969
DVI	*DVI* = *R*_*NIR*_−*R*_*RED*_	Richardson and Wiegand, 1977
PVI	$PVI=\left(R_{NIR}-10.489\times R_{RED}-6.604\right)/\sqrt{1+10.489^{2}}$	Huete et al., 1985
OSAVI	*OSAVI* = (1 + 0.16)(*R*_*NIR*_−*R*_*RED*_)/(*R*_*NIR*_ + *R*_*RED*_ + 0.16)	Rondeaux et al., 1996

Using the canopy spectral data of winter wheat, a regression estimation model with hyperspectral variables as independent variables and the chlorophyll content as dependent variables was established. Linear regression model was selected for all models:

(1) Y=a+bx

In this study, the coefficient of determination (*R*^2^) and the root mean square error (RMSE) were used to verify the linear regression model. The higher coefficient of determination R^2^ and the smaller RMSE indicated a more accurate estimation model.

(2) $$R^{2}=\frac{\sum_{i=1}^{n}{\left({\hat{y}}_{i}-{\bar{y}}_{i}\right)}^{2}}{\sum_{i=1}^{n}{\left(y_{i}-{\bar{y}}_{i}\right)}^{2}}$$

(3) $$RMSE=\sqrt{\frac{1}{n}\sum_{i=1}^{n}{\left({\hat{y}}_{i}-y_{i}\right)}^{2}}$$

Where ${\hat{y}}_{i}$ and y_*i*_ were the predicted values and measured values of the sample respectively, and i was the average value of the measured values of the sample, and n was the number of samples.

## Results

### Chlorophyll Content

The chlorophyll content in winter wheat under aCO_2_ and eCO_2_ in five growth stages was listed in Table 2. The chlorophyll content was lowest in the maturing stage and highest in the heading stage and varied under different CO_2_ treatments. At different growth stages, the effects of eCO_2_ on chlorophyll content of winter wheat were different. In booting and heading stage, eCO_2_ increased the chlorophyll content by 4.70–6.90%; in jointing, filling and maturity stage, eCO_2_ decreased the chlorophyll content by 2.80–18.20%. During the whole growth stage, the chlorophyll content under aCO_2_ was lower than that under eCO_2_ (Table 2).

**TABLE 2:** The chlorophyll content of winter wheat.

Data composition	Jointing	Booting	Heading	Filling	Maturity	*SD*	CV%
aCO_2_	48.04 ± 2.53^c^	55.50 ± 2.15^b^	57.42 ± 4.35^a^	55.32 ± 3.78^b^	44.28 ± 4.26^d^	7.33	14.70
eCO_2_	46.70 ± 3.53^d^	58.10 ± 4.97^b^	61.38 ± 3.75^a^	51.98 ± 2.51^c^	36.20 ± 7.36^e^	10.65	21.38

*SD is the standard deviation and the CV (%) is the coefficient of variation. Lowercase letters indicate significant levels (p < 0.05).*

### Canopy Spectral Reflectance

**FIGURE 1:**
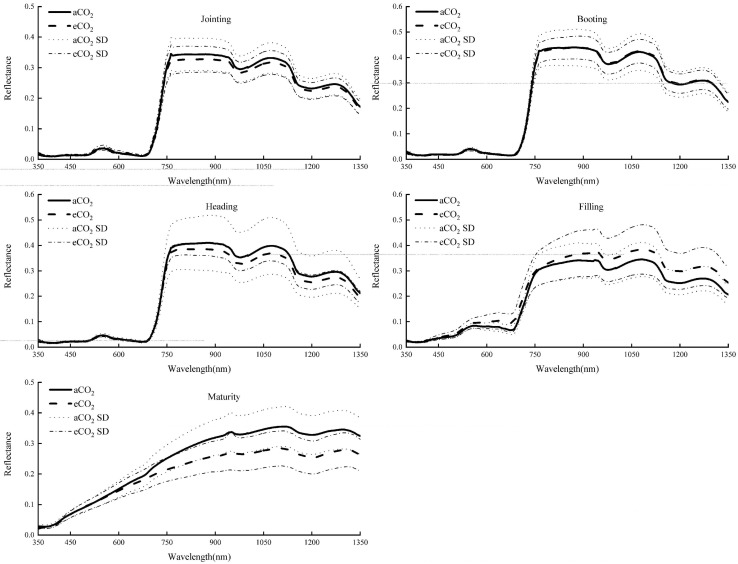
Spectral reflectance of winter wheat canopy at different growing stages under aCO_2_ and eCO_2_.

The original spectral band range was set at 350–1,350 nm and the canopy spectral reflectance under aCO_2_ and eCO_2_ at different growth stages was shown in Figure 1. In each growth stage, the reflectance showed similar trend under aCO_2_ and eCO_2_, with an absorption band around 500 nm, an obvious “green peak” around 550 nm, the minimum value around 680 nm, and a “red edge” within the band range of 680–760 nm.

The spectral reflectance of winter wheat canopy under eCO_2_ was slightly lower at jointing, heading and maturity stages, and was higher at filling stage than that at aCO_2_, especially in the wavelength range of 760–1,350 nm. In the booting stage, the spectral reflectance of the two treatments was similar. Among the five growth stages, the spectral reflectance at the booting stage was the highest, reaching about 0.45.

### Chlorophyll Content Estimation Models

#### The Red-Edge Position Model

**FIGURE 2:**
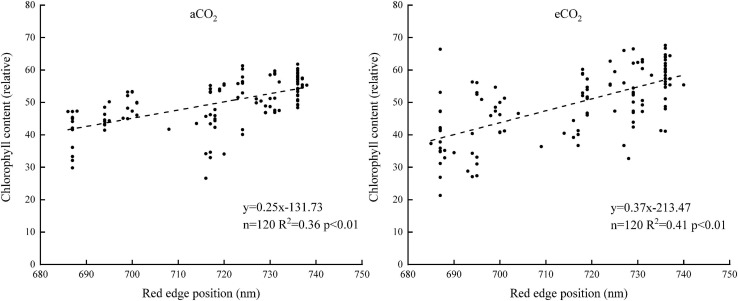
The relationship between the chlorophyll content and the red-edge positions.

**FIGURE 3:**
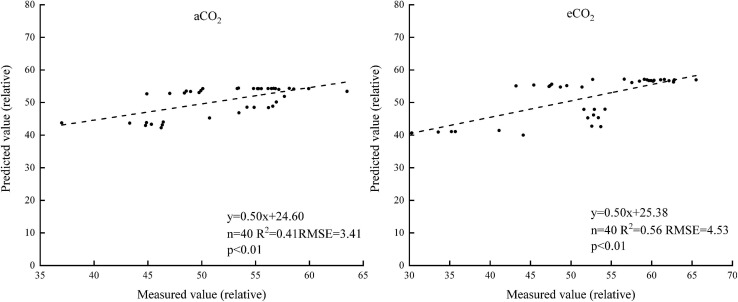
The measured and estimated values of the chlorophyll content based on the red-edge position model under aCO_2_ and eCO_2_.

Spectral reflectance rose rapidly at about 680 nm and slowly at about 760 nm. The band ranging between 680 and 760 nm was selected as the “red edge” spectrum. The linear regression model between the red edge position and the chlorophyll content was established to estimate chlorophyll content (Figure 2). The rest of the spectra and the chlorophyll content data were used to verify the model (Figure 3). The *R*^2^ of the model was 0.36 and 0.41 under aCO_2_ and eCO_2_, respectively (Figure 2). The estimation model based on the red edge location estimated chlorophyll content slightly better under eCO_2_ than under aCO_2_ (Figure 3).

#### The Sensitive Band Spectral Model

**FIGURE 4:**
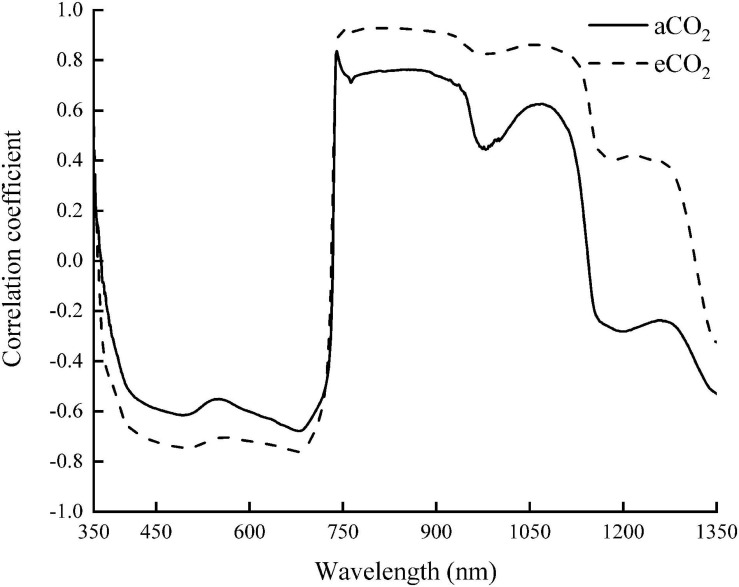
The correlations between the chlorophyll content and the spectral reflectance of winter wheat during the whole growing season.

The correlation coefficient of the spectral reflectance and chlorophyll content of winter wheat during the whole growth stage was analyzed (Figure 4). The correlation coefficient under aCO_2_ was higher than that under eCO_2_ between 350 and 1,350 nm. The canopy reflectance had the greatest correlation with the chlorophyll content at 740 and 749 nm under aCO_2_ and eCO_2_, respectively.

**FIGURE 5:**
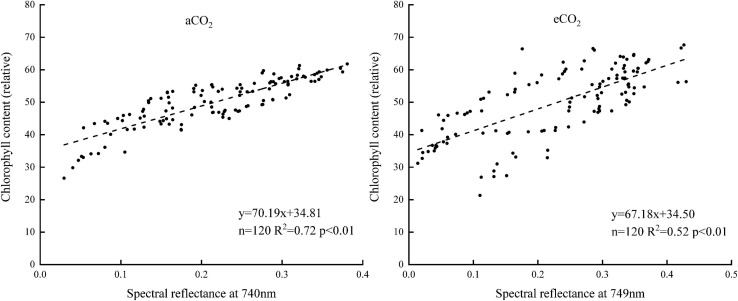
The relationship between the chlorophyll content and the sensitive bands position of the spectral reflectance.

**FIGURE 6:**
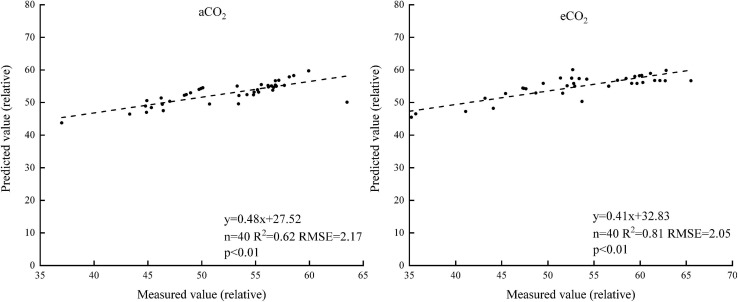
The measured and estimated values of chlorophyll content based on the sensitive band position model under aCO_2_ and eCO_2_.

The sensitive bands of 740 and 749 nm were then selected under aCO_2_ and eCO_2_ respectively, and the linear model between the spectral reflectance and chlorophyll content of the sensitive bands was established to estimate the chlorophyll content of winter wheat (Figure 5). The model was validated using the rest of the sampling data (Figure 6). The R^2^ of the linear model was 0.72 and 0.52 under aCO_2_ and eCO_2_, respectively (Figure 5) and the estimated values correlated well with the measured values of chlorophyll content under aCO_2_ and eCO_2_ (Figure 6).

#### The Spectral Index Model

**FIGURE 7:**
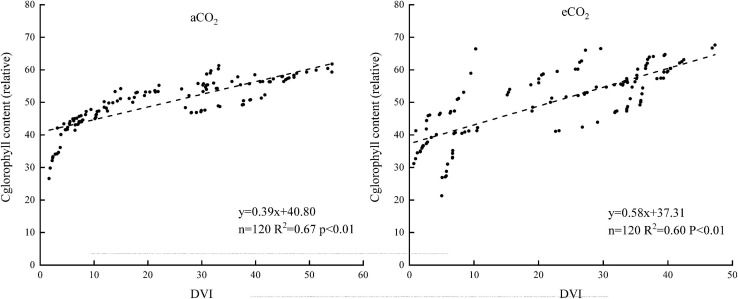
The relationship between the chlorophyll content and the DVI.

**FIGURE 8:**
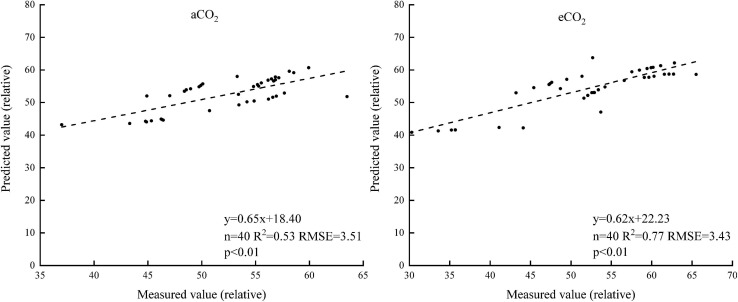
The measured and estimated values of the chlorophyll content based on the DVI model under aCO_2_ and eCO_2_.

Five different spectral indexes were extracted from the spectral reflectance curves (Table 2). Linear regression models of the five spectral indexes and chlorophyll contents of winter wheat were established. Under aCO_2_, the rank of *R*^2^ of the linear models was DVI > PVI > OSAVI > NDVI > RVI. Under eCO_2_, the rank of the *R*^2^ was DVI > OSAVI > NDVI > PVI > RVI (Table 3). The DVI based estimation models was established using half of the measured data (Figure 7) and validated using the rest of the sampling data (Figure 8). The *R*^2^ of the linear model established was 0.67 under aCO_2_ and 0.60 at eCO_2_ (Figure 7) and the estimated correlated well with the measured values of chlorophyll content under aCO_2_ and eCO_2_ (Figure 8).

**TABLE 3:** Estimation models of the chlorophyll content in wheat canopy based on different spectral indexes.

Treatment	Spectral index	Estimation equation	*R*^2^	Significance
aCO_2_	NDVI	y = 19.10x+38.22	0.56	*p* < 0.01
	RVI	y = 0.32x+46.38	0.21	*p* < 0.01
	DVI	y = 0.39x+40.80	0.67	*p* < 0.01
	PVI	y = 0.77x+34.28	0.57	*p* < 0.01
	OSAVI	y = 16.53x+38.23	0.56	*p* < 0.01
	NDVI	y = 27.83x+32.82	0.54	*p* < 0.01
	RVI	y = 0.50x+44.61	0.22	*p* < 0.01
eCO_2_	DVI	y = 0.58x+37.31	0.60	*p* < 0.01
	PVI	y = 0.71x+36.72	0.30	*p* < 0.01
	OSAVI	y = 24.04x+32.89	0.54	*p* < 0.01

## Discussion

In order to establish statistical models to study the relationship between the optical properties and chlorophyll content of winter wheat under elevated CO_2_ conditions, we measured the chlorophyll content and spectral reflectance in winter wheat canopy under aCO_2_ and eCO_2_ conditions throughout the growing season for 2 years. The effects of elevated CO_2_ on the chlorophyll content and spectral reflectance depended upon growing stages. The statistical models established in this study was effective under both ambient and elevated CO_2_ conditions.

Elevated CO_2_ increased the chlorophyll content of winter wheat at booting and heading stage, but decreased it at filling and maturity stage in this study. Elevated CO_2_ usually had a positive effect on the chlorophyll content, but the specific effect depended on treatment duration and different species (Long et al., 2004). The increase of CO_2_ concentration in the late growing stage might lead to the faster decline of chlorophyll concentration of wheat (Ommen et al., 1999). In this study, the senescence of winter wheat under eCO_2_ was faster than that under aCO_2_ and the chlorophyll content was decreased under eCO_2_ at the later growing stages. The overall shapes of spectral curves did not change throughout the growing season, except in the maturity stage, the curves flattened due to the senescence of the leaves. The effect of elevated CO_2_ on the spectral curves varied at different growing stages, with no impact in the earlier jointing and boosting stages, positive impact in the filling and negative impact in the heading and maturity stages. Though elevated CO_2_ changed the maximum reflectance, it did not change the overall shape of the spectral curves of winter wheat at all the growing stages. The results were consistent with previous studies where the shapes of soybean canopy spectral curve did not change under different CO_2_ treatments (Gray et al., 2010) and O_3_ concentrations (Campbell et al., 2007).

Red edge position, sensitive band and vegetation index were effective means to retrieve crop chlorophyll content from the spectral curves (Dou et al., 2018; Kasim et al., 2018; Wang et al., 2019). Previous studies had shown that the position and reflectance of red edge were highly correlated with chlorophyll content of plant leaves and could be used as an indicator of chlorophyll content (Filella and Penuelas, 1994; Gitelson et al., 1996). The current study showed that the reflectance at the 680–740 nm wavelengths had a positive relationship with the content of chlorophyll under both ambient and elevated CO_2_.

Sensitive bands could be used to calculate spectral indexes, which were sensitive to the difference of chlorophyll concentration in plant canopy (Hunt et al., 2011). The current results showed that the sensitive bands at 740 and 749 nm wavelength correlated with the chlorophyll content most, under aCO_2_ and eCO_2_, respectively (Figures 4–6), even though the established model fit slightly better under aCO_2_ than at eCO_2_ conditions. Vegetation indexes calculated from hyperspectral remote sensing technology had long been used to monitor the chlorophyll content of vegetation leaves (Meng et al., 2012; Guo et al., 2020). Among the five tested vegetation indexes, the DVI based model simulated the chlorophyll content best under both aCO_2_ and eCO_2_ conditions and the model using overall data from both the CO_2_ treatments gave similar results (results not shown). Though the methods tested in the study proved effective to simulate winter wheat chlorophyll content under different CO_2_ conditions, further investigations on how the spectral reflectance correlates with other biochemical contents and biophysical processes are still urgently needed for the purpose of guiding crop management and monitoring crop growth status in the future climate change situations.

In conclusion, the hyperspectral estimation models based on the red edge position, sensitive band and DVI vegetation index could all simulate the chlorophyll content of winter wheat. The accuracy of vegetation index and sensitive bands based models was higher than that of the red edge position model. The results suggested that the hyperspectral measurement can be effectively used to estimate the chlorophyll content under both aCO_2_ and eCO_2_ conditions and different equations should be established at specific CO_2_ growing conditions based on the methods chosen. The findings in the study were useful in providing hyperspectral methods to monitor the growth status of winter wheat in the future global change situations.

## Data Availability Statement

The raw data supporting the conclusions of this article will be made available by the authors, without undue reservation.

## Author Contributions

DW designed and came up with the idea of the experiment. YC conducted the experiment, analyzed data, and wrote the manuscript. YM and HW helped in the field experiment and provided critical feedbacks on the manuscript. All authors contributed to the article and approved the submitted version.

## Conflict of Interest

The authors declare that the research was conducted in the absence of any commercial or financial relationships that could be construed as a potential conflict of interest.
